# Insights into the Cultured Bacterial Fraction of Corals

**DOI:** 10.1128/mSystems.01249-20

**Published:** 2021-06-22

**Authors:** Michael Sweet, Helena Villela, Tina Keller-Costa, Rodrigo Costa, Stefano Romano, David G. Bourne, Anny Cárdenas, Megan J. Huggett, Allison H. Kerwin, Felicity Kuek, Mónica Medina, Julie L. Meyer, Moritz Müller, F. Joseph Pollock, Michael S. Rappé, Mathieu Sere, Koty H. Sharp, Christian R. Voolstra, Nathan Zaccardi, Maren Ziegler, Raquel Peixoto

**Affiliations:** a Aquatic Research Facility, Environmental Sustainability Research Centre, University of Derby, Derby, United Kingdom; b Federal University of Rio de Janeiro, Rio de Janeiro, Brazil; c Institute for Bioengineering and Biosciences (iBB), University of Lisbon, Lisbon, Portugal; d Instituto Superior Técnico (IST), University of Lisbon, Lisbon, Portugal; e Department of Energy, Joint Genome Institute and Lawrence Berkeley National Laboratory, Berkeley, California, USA; f Gut Microbes and Health, Quadram Institute Bioscience, Norwich, United Kingdom; g College of Science and Engineering, James Cook University, Townsville, Australia; h Australian Institute of Marine Science, Townsville, Australia; i Department of Biology, University of Konstanz, Konstanz, Germany; j School of Environmental and Life Sciences, The University of Newcastle, Ourimbah, NSW, Australia; k Centre for Marine Ecosystems Research, Edith Cowan University, Joondalup, WA, Australia; l Department of Biology, McDaniel College, Westminster, Maryland, USA; m College of Public Health, Medical and Veterinary Sciences, James Cook University, Townsville, Australia; n Department of Biology, Pennsylvania State University, University Park, Pennsylvania, USA; o Soil and Water Sciences Department, Genetics Institute, University of Florida, Gainesville, Florida, USA; p Faculty of Engineering, Computing and Science, Swinburne University of Technology Sarawak Campus, Kuching, Sarawak, Malaysia; q Hawaii and Palmyra Programs, The Nature Conservancy, Honolulu, Hawaii, USA; r Hawaii Institute of Marine Biology, University of Hawaii, Kaneohe, Hawaii, USA; s Department of Biology and Marine Biology, Roger Williams University, Bristol, Rhode Island, USA; t Department of Animal Ecology and Systematics, Justus Liebig University Giessen, Giessen, Germany; u Red Sea Research Center (RSRC), Division of Biological and Environmental Science and Engineering (BESE), King Abdullah University of Science and Technology (KAUST), Thuwal, Saudi Arabia; Lawrence Berkeley National Laboratory; University of North Carolina at Chapel Hill; Farmingdale State College

**Keywords:** symbiosis, holobiont, metaorganism, cultured microorganisms, coral, probiotics, beneficial microbes, genomes, symbiosis

## Abstract

Bacteria associated with coral hosts are diverse and abundant, with recent studies suggesting involvement of these symbionts in host resilience to anthropogenic stress. Despite their putative importance, the work dedicated to culturing coral-associated bacteria has received little attention. Combining published and unpublished data, here we report a comprehensive overview of the diversity and function of culturable bacteria isolated from corals originating from tropical, temperate, and cold-water habitats. A total of 3,055 isolates from 52 studies were considered by our metasurvey. Of these, 1,045 had full-length 16S rRNA gene sequences, spanning 138 formally described and 12 putatively novel bacterial genera across the *Proteobacteria*, *Firmicutes*, *Bacteroidetes*, and *Actinobacteria* phyla. We performed comparative genomic analysis using the available genomes of 74 strains and identified potential signatures of beneficial bacterium-coral symbioses among the strains. Our analysis revealed >400 biosynthetic gene clusters that underlie the biosynthesis of antioxidant, antimicrobial, cytotoxic, and other secondary metabolites. Moreover, we uncovered genomic features—not previously described for coral-bacterium symbioses—potentially involved in host colonization and host-symbiont recognition, antiviral defense mechanisms, and/or integrated metabolic interactions, which we suggest as novel targets for the screening of coral probiotics. Our results highlight the importance of bacterial cultures to elucidate coral holobiont functioning and guide the selection of probiotic candidates to promote coral resilience and improve holistic and customized reef restoration and rehabilitation efforts.

**IMPORTANCE** Our paper is the first study to synthesize currently available but decentralized data of cultured microbes associated with corals. We were able to collate 3,055 isolates across a number of published studies and unpublished collections from various laboratories and researchers around the world. This equated to 1,045 individual isolates which had full-length 16S rRNA gene sequences, after filtering of the original 3,055. We also explored which of these had genomes available. Originally, only 36 were available, and as part of this study, we added a further 38—equating to 74 in total. From this, we investigated potential genetic signatures that may facilitate a host-associated lifestyle. Further, such a resource is an important step in the selection of probiotic candidates, which are being investigated for promoting coral resilience and potentially applied as a novel strategy in reef restoration and rehabilitation efforts. In the spirit of open access, we have ensured this collection is available to the wider research community through the web site http://isolates.reefgenomics.org/ with the hope many scientists across the globe will ask for access to these cultures for future studies.

## INTRODUCTION

In recent years, the concept of the metaorganism or holobiont, which defines the associations formed by a host organism and its microbiome (1–3), has become a cornerstone of biology (4). Scleractinian corals are an excellent example of host-microbe associations, as they build reefs through close symbiotic interactions between the host modular animal, its endosymbiotic dinoflagellates (*Symbiodiniaceae*), and an array of other microbial partners, including bacteria, archaea, and fungi (3, 4). The bacterial taxa associated with corals can vary between coral species and geographical origin, though often there are patterns in the community structure that link microbial and coral taxa (5, 6). Many original discoveries on the importance of coral-associated bacteria and their interactions with the coral host were made using culture-based methods (7, 8). However, the majority of recent studies exploring the importance of coral-associated microbes have focused on the use of cultivation-independent approaches, based on 16S rRNA gene amplicon sequencing (9) and, more recently, shotgun metagenomics (10, 11). Such methods are central in identifying what bacteria are associated with corals and how their metabolic and functional potential contribute to holobiont health and response to environmental conditions (9, 12–14). However, the bacterial metabolic pathways that interact with the host and respond to environmental changes are often best understood using culture-based approaches (15). This is particularly relevant because metagenomic information gives insights into potential functional traits and other cellular traits only, and often environmental changes have pleiotropic effects on holobiont physiology that are impossible to grasp using metagenomics alone (16–19).

Inherently, culture-based approaches retrieve only a small fraction of the total bacterial diversity within any given environment, a phenomenon known as the “great plate anomaly” (20–22). Often however, it is not a case of being “unculturable” but of not yet knowing the (range of) conditions needed to culture specific microorganisms (23). Cultivating host-associated microorganisms can be challenging, as their nutrient requirements and cross-feeding networks are often unknown (24). In addition, many “environmental” microorganisms grow very slowly (in contrast to clinical isolates), and are not adapted to or capable of growing on commonly used nutrient-rich media, and are outcompeted by copiotrophic bacteria (25, 26). To counter this, at least to some degree, recent studies have implemented novel and alternative culture-based methods to retrieve a higher proportion of the bacterial diversity present in any given sample (24, 27, 28), and these approaches have also been applied to corals (29–31).

Organismal, growth form, and tissue complexity create unique microenvironments that are thought to contribute to the high bacterial diversity often seen in corals (32–34). The diverse coral bacteriome plays an integral role in the balance between health and disease of the coral holobiont (35, 36) and represents a valuable source of biotechnological products (37, 38). Disalvo (39) was perhaps the first to isolate bacteria from coral in 1969, recovering strains from the skeletal regions of Porites lobata, followed by Ducklow and Mitchell (40) who reported on bacteria isolated from mucus of Porites astreoides and two octocoral species 10 years later. Microbe-mediated diseases have also been well documented as driving declines in reef health, especially throughout the Caribbean for example (41). This has fostered a great interest in understanding coral disease causative agents, stimulating cultivation efforts of coral-associated bacteria (42–44). For example, Kushmaro et al. (45) isolated a bacterium that caused bleaching of the coral Oculina patagonica, and many subsequent studies have implicated vibrios in coral disease causation (46–48)—although it should be noted that coral bleaching is not typically considered a disease and is ascribed to dysbiosis of the coral host and associated *Symbiodiniaceae* (49). Regardless, many of these studies focused on targeted isolation and conducted reinfection studies to satisfy Koch’s postulates, with varying success (reviewed in reference 50).

Counter to the notion of pathogenicity of certain bacteria, growing evidence underlines the key role secondary metabolites produced by (beneficial) bacteria have on host health (35, 51–54). For instance, Ritchie (55) was among the first to demonstrate that mucus-associated bacteria from healthy colonies inhibit the growth of potential pathogens. Subsequent studies revealed high antimicrobial activity among culturable coral-associated bacteria, with up to 25% of the isolates producing antimicrobial compounds (56). Kuek et al. (57) showed a strong link between observed antibiotic activity in well diffusion assays and existence of polyketide synthase (PKS) and/or nonribosomal peptide synthetase (NRPS) genes in the bacterial isolates. More recently, Raina et al. (17) found that the antimicrobial compound tropodithietic acid (TDA) was produced by the coral-associated bacterium *Pseudovibrio* sp. and subsequent studies found that *Pseudovibrio* species harbor several biosynthetic gene clusters for the synthesis of bioactive compounds (58, 59).

**FIG 1:**
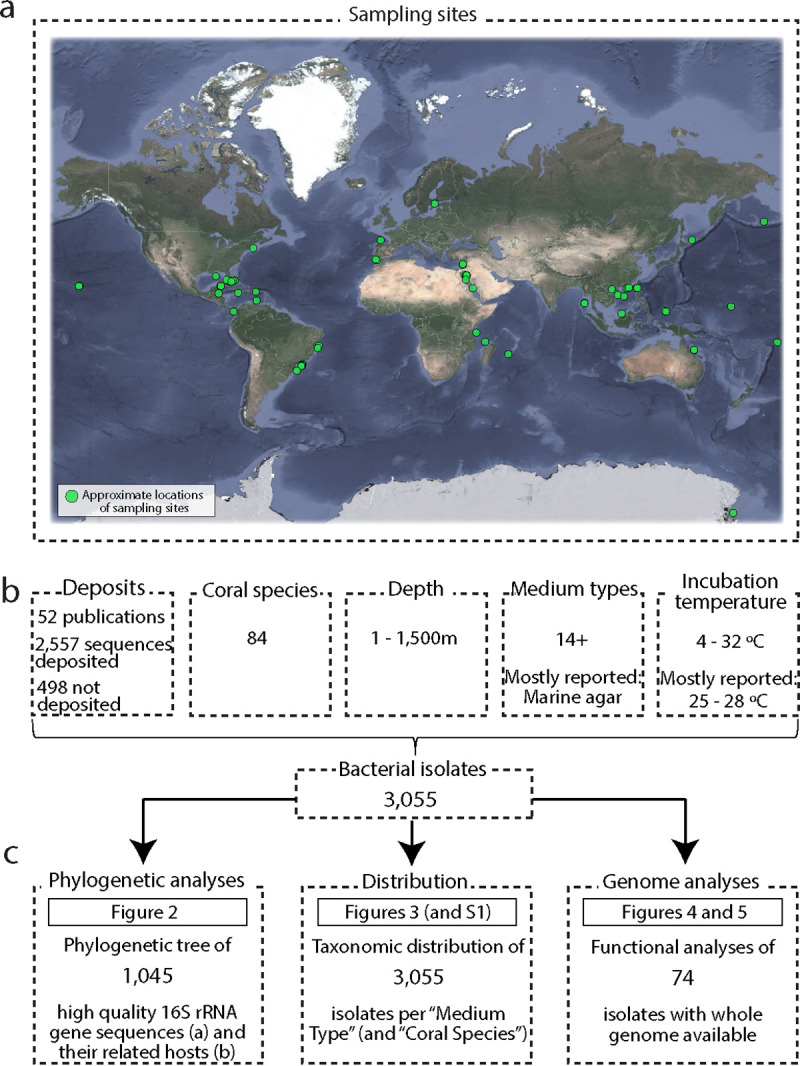
Overview of the data detailed in this article. (a) Sampling sites of the coral species used as isolation sources. Map data © 2020 Google. (b) Data summary recovered from the publications and accession numbers available in data banks. (c) Overview of the analyses performed in the current article using the available isolates.

Bacterial isolates from corals represent an invaluable resource for assessing the virulence of potential pathogens, and for applying classical clinical approaches to elucidate disease etiology (60). Beneficial traits that bacteria may provide to coral holobiont functioning can also be elucidated using pure bacterial cultures (10, 18). Bacteria isolated from corals can also be used as probiotics to facilitate host health (61, 62), and such approaches have been proposed to promote coral resilience in the face of environmental stress. For example, Rosado et al. (53) showed that application of so-called “beneficial microorganisms for corals” (or BMCs) increases the resilience of the coral to temperature stress and pathogen challenge. However, despite the demonstrated importance of BMCs (63), a centralized and curated collection of isolates obtained from corals and their associated genetic information does not currently exist. Moreover, many culture-based studies often focus on relatively few bacteria (targeted for pathogenic agents for example), meaning a large-scale comparison of which bacterial isolates can be cultured and their genetic information is currently missing. Here, we sought to centralize and curate the current cultured fraction of coral bacteria by combining published data with unpublished collections from around the world (Fig. 1). Without doubt, some studies and culture collections will have been missed in this first compilation; however, our aim was to start building a resource that can be built upon. To highlight the importance of such a collection, we explore the relationships between the isolated bacteria, the host origin, and the media utilized for growth. Further, a total of 74 genomes of cultured coral bacteria, 36 of which are available in public databases and 38 of which are presented in this study for the first time, were investigated to infer potential genetic signatures that may facilitate a host-associated lifestyle. Finally, alternative ways and improvements for the isolation of bacterial groups not yet recovered from corals (including the specific targeting of obligate symbionts) are discussed. This study provides the most comprehensive synthesis of the cultured bacterial fraction of the coral holobiont thus far.

## RESULTS

### Phylogenetic analysis of culturable coral-associated bacteria.

**FIG 2:**
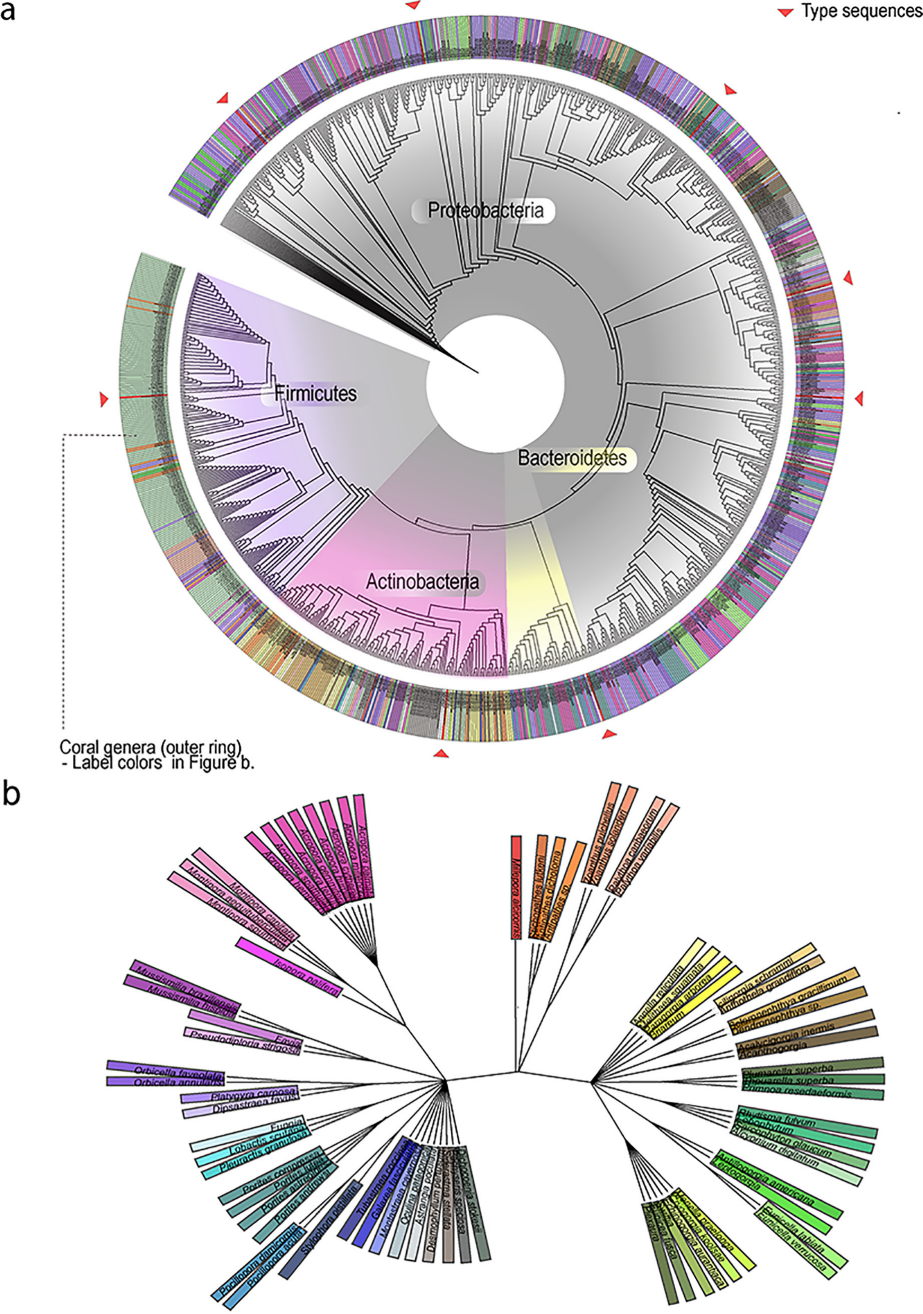
Phylogenetic trees of bacterial strains and coral species. (a) 16S rRNA gene-based phylogenetic inference of 1,045 coral-associated bacterial isolates, plus eight type strains (marked with red arrowheads) representing the species Vibrio alginolyticus, Vibrio bivalvicida, Pseudoalteromonas aestuariivivens, Pseudomonas guariconensis, Massilia namucuonensis, Vibrionimonas magnilacihabitans, Mycetocola tolaasinivorans, and Bacillus subtilis. The colors on the outer ring refer to the coral genus from which the bacteria were isolated, and the background colors in the center refer to the bacteria phyla. (b) Phylogenetic tree of the species of corals used in this study produced via (https://www.ncbi.nlm.nih.gov/Taxonomy/CommonTree/wwwcmt.cgi). The label colors used to identify the genera are linked to the outer ring of Fig. 2A.

To define the relationships and a taxonomic overview of the groups of coral-associated bacteria isolated from around the world, published and unpublished data sets were interrogated, identifying 3,055 cultured coral-associated bacteria, for which 1,045 high-quality full-length 16S rRNA gene sequences are available (Fig. 2a). Altogether, these data indicate that bacteria from at least 138 genera can be cultured from corals using a variety of different media (12 defined commercial media and various bespoke custom media). While most isolates belong to the phylum *Proteobacteria* (72% of those cultured), strains from *Firmicutes* (14%), *Actinobacteria* (10%), and *Bacteroidetes* (5%) were also recovered. The genera *Ruegeria*, *Photobacterium*, Pseudomonas, *Pseudoalteromonas*, *Vibrio*, *Pseudovibrio*, and *Alteromonas* were commonly isolated across studies (see Tables S1, S3, and S4 in the supplemental material). Of 43 genera identified as putative beneficial microbes (proposed in current literature; see examples and references in Table S4), 58% (i.e., 25 isolates) have been shown to be culturable and are represented in this collection (Table S4). Most of the isolates that have been cultured from diseased corals belong to the family *Vibrionaceae* (*Proteobacteria*). However, it should be noted that many of the studies reporting *Vibrionaceae* focused on a targeted approach to isolate these bacteria. Among the isolates from the phylum *Proteobacteria*, 25.5% were associated with diseased coral colonies, as were 7.4% of the isolates belonging to the phylum *Bacteroidetes*. *Firmicutes* and *Actinobacteria* had the lowest cultivation success from diseased corals, with 5.0% and 0.7%, respectively. Although the majority of the isolates were matched with other representatives in GenBank, 12 were highly divergent with low similarity to known isolates, suggesting that they may be novel genera.

10.1128/mSystems.01249-20.2 TABLE S1 Meta-data for the coral-associated bacteria isolated and/or discussed as part of this study, including GenBank accession numbers, isolation source, media, and 16S sequence for example. References and link to the original paper where some of these isolates were originally described has also been included where relevant. Download Table S1, XLSX file, 0.8 MB. Copyright © 2021 Sweet et al. 2021 Sweet et al. https://creativecommons.org/licenses/by/4.0/ This content is distributed under the terms of the Creative Commons Attribution 4.0 International license.

10.1128/mSystems.01249-20.4 TABLE S3 Information on the coral species, number of cultured coral-associated bacteria identified from this analysis/study, along with the number of operational taxonomic units (OTUs) described for the coral species via next generation sequencing (if known), and relevant references if available. There is also the calculation for the percentage culturable fraction assessed by simply dividing the number of bacteria cultured for the specific coral over the mean OTUs recorded from published literature. This should be taken rather objectively, as the culture effort was not standardized in any way. Download Table S3, XLSX file, 0.02 MB. Copyright © 2021 Sweet et al. 2021 Sweet et al. https://creativecommons.org/licenses/by/4.0/ This content is distributed under the terms of the Creative Commons Attribution 4.0 International license.

10.1128/mSystems.01249-20.5 TABLE S4 Proposed key, core, or functionally important bacteria with references (scientific paper) where their importance is discussed in greater detail. Download Table S4, XLSX file, 0.01 MB. Copyright © 2021 Sweet et al. 2021 Sweet et al. https://creativecommons.org/licenses/by/4.0/ This content is distributed under the terms of the Creative Commons Attribution 4.0 International license.

### Taxonomic composition of bacterial isolates by culture medium.

**FIG 3:**
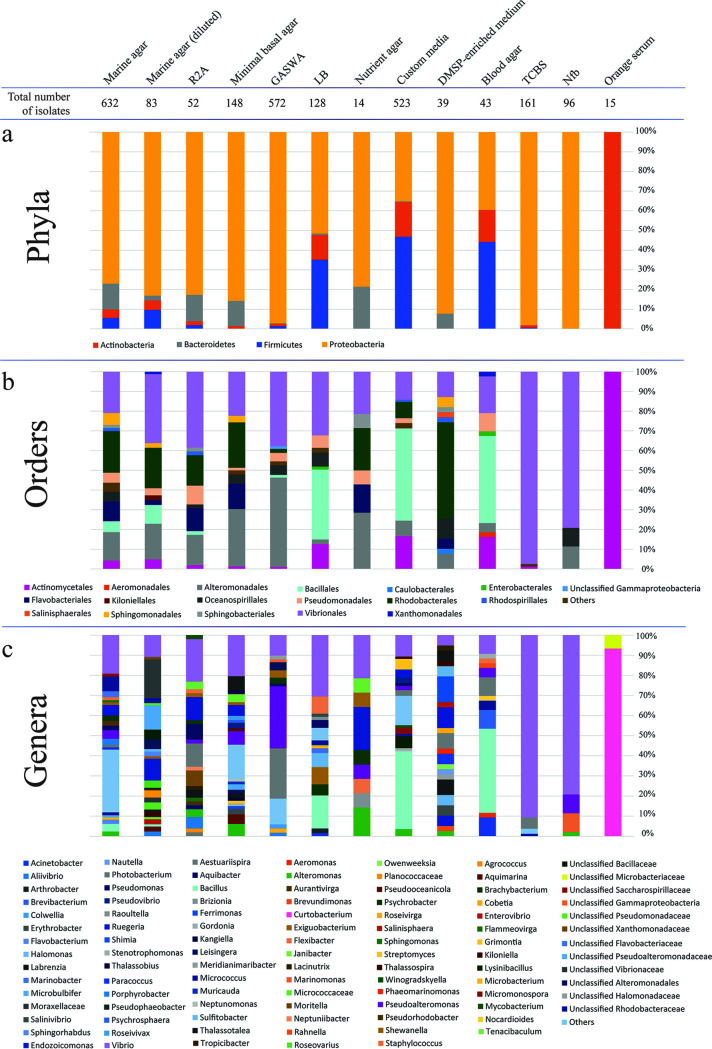
Phylum (a), order (b), and genus (C) level profiles of coral-associated bacteria isolated from each type of culture medium. Taxa (i.e., orders and genera) representing less than 1% of the total percentage of isolates were pulled together and classified as “Others.”

The taxonomic patterns of the cultured bacterial strains at the phylum, order, and genus levels varied according to the type of medium used to isolate them (Fig. 3). Marine agar (MA) (including its diluted versions) was the most commonly utilized medium across studies and supported the growth of 715 distinct isolates collectively. Bacterial isolates belonging to the families *Vibrionaceae*, *Alteromonadaceae*, *Pseudoalteromonadaceae*, *Rhodobacteraceae*, *Flavobacteriaceae*, and *Micrococcaceae* could all be isolated from MA from a diverse set of coral species. The next most productive nonselective medium was glycerol artificial seawater agar (GASWA), which supported the growth of 572 distinct isolates, while a variety of “custom” media from different laboratories supported the growth of 523 isolates. Interestingly, the latter collection of media, i.e., the custom variants (along with blood agar specifically), favored the retrieval of *Firmicutes* (46.8% of isolates) and *Proteobacteria* representatives (35.2%) at the expense of *Actinobacteria* species (17.6%). In contrast, media commonly deployed to sample a wider bacterial diversity, such as marine agar, favored the growth of several *Proteobacteria* species, usually affiliated with diverse clades within the *Alphaproteobacteria* and *Gammaproteobacteria* classes (Fig. 2 and 3). Curiously, use of thiosulfate-citrate-bile salts-sucrose medium (TCBS) supported the growth of manifold bacterial lineages across the four phyla documented in this study, including *Micrococcus* and *Photobacterium* for example, despite its presumed selectivity for *Vibrio* species.

Bacteria belonging to the phylum *Proteobacteria* (the dominant isolates captured in this study, 72%) could be retrieved from nearly all cultivation media and conditions examined, according to the design and scope of the study (Fig. 2). Members of other abundant phyla, i.e., *Firmicutes*, *Actinobacteria*, and *Bacteroidetes*, also appeared to be cultured on most media (Fig. 3a). Orange serum agar seemed to be selective for *Actinobacteria* (Table S1). The media MA, R2A, and minimal basal agar shared a very similar pattern at the order level, all yielding similar proportions of members from the orders *Vibrionales*, *Rhodobacterales*, *Pseudomonadales*, *Flavobacteriales*, and *Actinomycetales* (Fig. 3). Likewise, LB, blood agar, and the “custom” media shared similar patterns, which included the orders *Vibrionales*, *Pseudomonadales*, *Bacillales*, *Alteromonadales*, and *Actinomycetales* (Fig. 3b). At the genus level, no immediate patterns seemed to be shared among the media (Fig. 3c). The highest number of unique isolates identified to genus level was obtained from MA, which had 115 unique isolates, followed by 55 isolates from custom media, 48 from minimal basal media, and 47 from GASWA (Table S1). However, when dividing the number of different genera by the total number of isolates in each medium, the normalized ratios show that nutrient agar (0.64), followed by dimethylsulfoniopropionate (DMSP)-enriched media (0.54) and R2A (0.4), supported the growth of higher bacterial diversity. Conversely, lowest bacterial diversities were found on TCBS (0.04), Nfb (0.04), and GASWA (0.08). The normalized ratios for each medium (considering all the isolates analyzed here) can be found in Table S1.

### Functional genomics of coral bacterial isolates.

A total of 74 cultured coral-associated bacteria had full or draft genomes available; 36 genomes were accessible as of February 2020, with a further 38 genomes now available from this study (Table S2). The genome sizes ranged from 2.71 Mb in *Erythrobacter* sp. strain A06_0 (associated with the scleractinian coral Acropora humilis) with only 2,669 coding sequences (CDSs), to 7.28 Mb in Labrenzia alba (synonym Roseibium album) EL143 (associated with the octocoral Eunicella labiata) with 7,593 CDSs (Table S2). The mean and median genome size was 4.77 Mb and 4.71 Mb, respectively. The average GC content of these genomes was 53%, with the lowest GC content (32.9%) found in Aquimarina megaterium strain EL33 (isolated from E. labiata), and the highest GC content (71.4%) found in Janibacter corallicola strain NBRC 107790 (from Acropora gemmifera).

10.1128/mSystems.01249-20.3 TABLE S2 Basic information of 74 coral bacterial isolates with their genomes sequences available in public databases (NCBI; IMG/DOE-JGI). Download Table S2, XLSX file, 0.03 MB. Copyright © 2021 Sweet et al. 2021 Sweet et al. https://creativecommons.org/licenses/by/4.0/ This content is distributed under the terms of the Creative Commons Attribution 4.0 International license.

**FIG 4:**
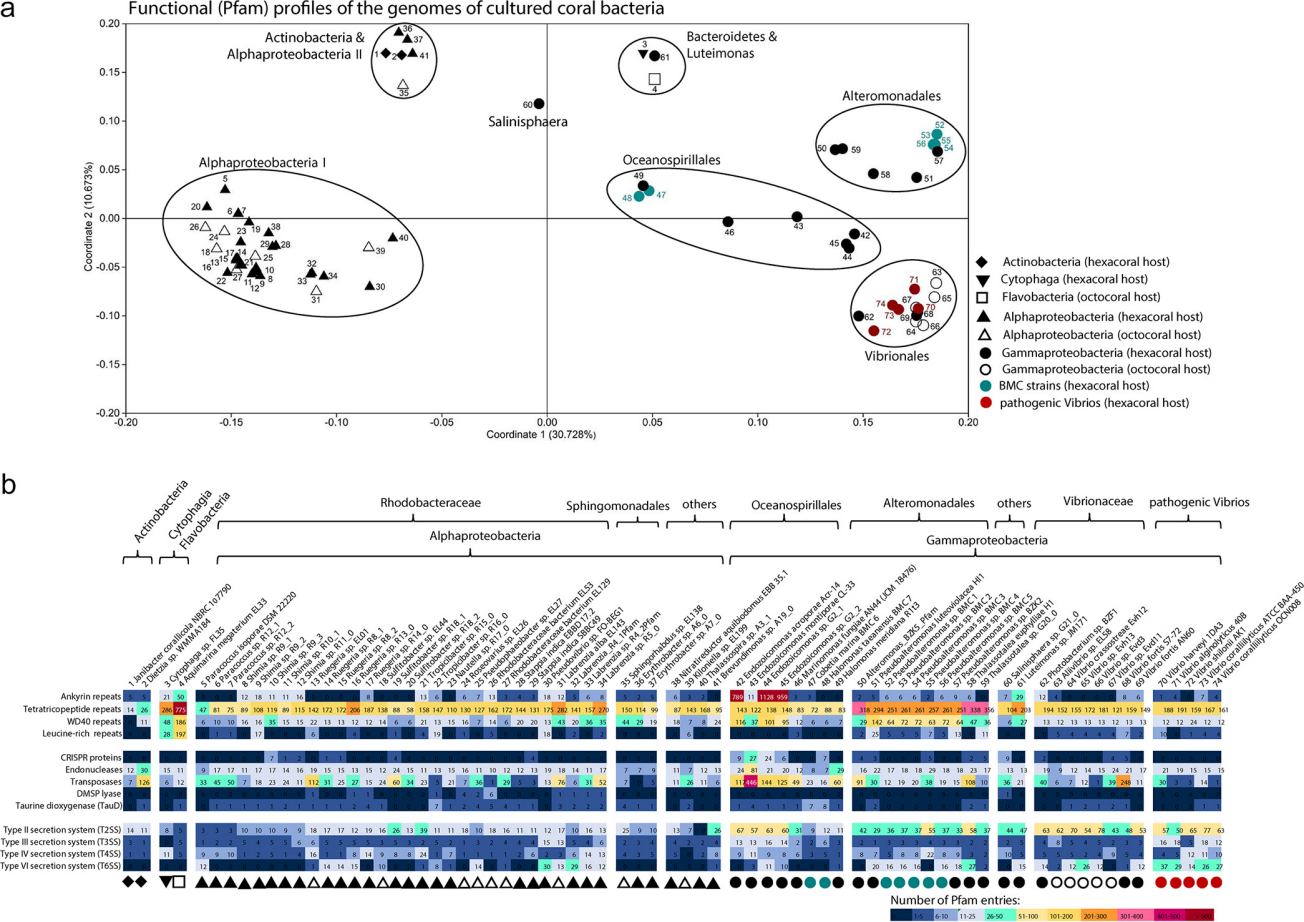
Functional analysis of 74 genomes of cultured coral bacteria according to their protein family (Pfam) profiles. Principal coordinate analysis (PCoA) was performed on the Pfam profiles using the Bray-Curtis similarity matrix calculated from Hellinger-transformed abundance data (a). The ordination is shown in eigenvalue-scale. Symbol shapes indicate the taxonomic class of each genome and the host origin (filled symbols for scleractinian corals; open symbols for octocorals). In addition, BMC bacteria are highlighted in cyan blue, while typical coral pathogens are highlighted in dark red. Isolate numbers (as in panel b) are given next to each symbol. The number of CDSs assigned to Pfam entries related to eukaryote-like proteins “ELPs” (i.e., ankyrin-, tetratricopeptide-, WD40- and leucine-rich repeats) and other features involved in host-microbe interactions are highlighted in the table below (b). The color code from dark blue to dark red reflects an increase in the number of CDSs related to each function. ELPs, CRISPR proteins, endonucleases, transposases, and secretion systems were each represented by more than one Pfam entry across the data set. The CDS counts of these functionally belonging Pfams were summed. The number of Pfams that contributed to each function were as follows: ankyrin repeats, 5 Pfam entries; tetratricopeptide repeats, 21 Pfam entries; WD40 repeats, 6 Pfam entries; leucine-rich repeats, 8 Pfam entries; CRISPR proteins, 21 Pfam entries; endonucleases, 42 Pfam entries; transposases, 37 Pfam entries; T2SS, 17 Pfam entries; T3SS, 19 Pfam entries; T4SS, 15 Pfam entries; T6SS, 18 Pfam entries (see Table S5 in the supplemental material for Pfam identifiers [IDs] and names). In the case of taurine and dimethylsulfoniopropionate (DMSP) catabolism, only one Pfam entry (PF02668.16 and PF16867.5) was found, respectively.

Multivariate analysis, based on protein family (Pfam) profiles (Fig. 4a), unsurprisingly showed that the genomes grouped mostly according to their (class level) taxonomic affiliations (permutational multivariate analysis of variance [PERMANOVA], *F *= 11.55, *P* = 0.0001). Exceptions were the two *Actinobacteria* and two *Bacteroidetes* genomes, which clustered with four *Alphaproteobacteria* genomes of the order *Sphingomonadales* and *Caulobacterales* and the *Luteimonas* sp. strain JM171 (*Gammaproteobacteria*) genome, respectively. However, this is likely a reflection of the very low number of genomes available from coral-associated *Actinobacteria* and *Bacteroidetes*, rather than a significant functional overlap between the two phyla. Interestingly, a PERMANOVA analysis performed on the Pfam profiles of the *Vibrionales* genomes revealed that the five *Vibrio* genomes from known pathogens were significantly different from all nonpathogenic *Vibrionaceae* strains (*P* = 0.0006, df = 1, *F *= 1.829) (Table S5).

10.1128/mSystems.01249-20.6 TABLE S5 Functional profiling of the genomes of 74 coral-associated bacteria according to Protein families (Pfam) annotations using the web-based service WebMGA (S. Wu, Z. Zhu, L. Fu, B. Niu, W. Li, BMC Genomics 12:444, 2011, https://dx.doi.org/10.1186/1471-2164-12-444). Download Table S5, XLSX file, 2.1 MB. Copyright © 2021 Sweet et al. 2021 Sweet et al. https://creativecommons.org/licenses/by/4.0/ This content is distributed under the terms of the Creative Commons Attribution 4.0 International license.

Functions that potentially have a role in host-microbe interactions, such as proteins containing eukaryote-like domains involved in host-symbiont recognition (11, 64, 65), secretion systems potentially important for host colonization, and biosynthetic gene clusters encoding secondary metabolites were investigated across the isolates (Fig. 4b). Eukaryote-like repeat proteins (ELPs), such as ankyrin repeats, WD40 repeats, tetratricopeptide repeats, and leucine-rich repeats, are widely existing protein motifs which mediate protein-protein interactions. They can be found in all domains of life but are most common in eukaryotes (66–69). The *Endozoicomonas* strains G2_1, G2_2, and Acr-14 had the highest number of ankyrin repeats (>789), and high numbers of WD40 repeats (between 37 and 116). In contrast, ankyrin repeats were absent or only present in low numbers in all *Vibrio* strains. *Alteromonadales* strains (including the *Pseudoalteromonas* BMCs), had high numbers of tetratricopeptide (>250) and 29 to 142 WD40 repeats. The strain with the overall highest number of eukaryote-like repeat protein-related entries (1,367 repeats) was *Endozoicomonas* sp. strain G2_01 from Acropora cytherea, closely followed by the octocoral associate Aquimarina megaterium EL33 (class *Flavobacteria*) (1,208 repeats). Endozoicomonas montiporae strain CL-33 displayed the highest number of domains related to antiviral defense mechanisms, such as CRISPR proteins and endonucleases, which are known to be enriched in the microbiomes of marine sponges (65, 70) and healthy octocorals (11). Further, 49 out of the 74 genomes assessed harbored the TauD (PF02668) gene. TauD is involved in the degradation of host-derived taurine (an amino-sulfonic acid widely distributed in animal tissue) into sulfide which is then assimilated into microbial biomass (71–73). An elevated number of TauD-encoding CDSs was found in the two BMC strains Cobetia marina BMC6 and Halomonas tateanensis BMC7, both isolated from Pocillopora damicornis. Further, several isolates (*N *= 11) of the *Rhodobacteraceae* family (*Alphaproteobacteria*) contained CDSs involved in dimethylsulfoniopropionate (DMSP) degradation, potentially contributing to sulfur cycling in corals.

Among secretion systems, type II (T2SS), III (T3SS), IV (T4SS), and VI (T6SS), known to be involved in host colonization (74), horizontal gene transfer (75), or interbacterial antagonism and/or virulence (76), dominated the genomes of coral-associated bacteria. We found a high number of entries related to T2SS in the *Gammaproteobacteria* associates, particularly in the *Endozoicomonas* and *Vibrio* genomes (see reference 77 for roles of the T2SS in symbiosis and pathogenicity). The *Vibrionales* genomes were further characterized by an elevated number of T6SS-related Pfam domains, whereby the five pathogenic *Vibrio* strains encoded a significantly higher number of T6SS domains (mean of 27 T6SS domains in CDSs) than the six nonpathogenic *Vibrio* strains (mean of 10 T6SS domains in CDSs; Mann-Whitney U-test, *P* = 0.0126).

**FIG 5:**
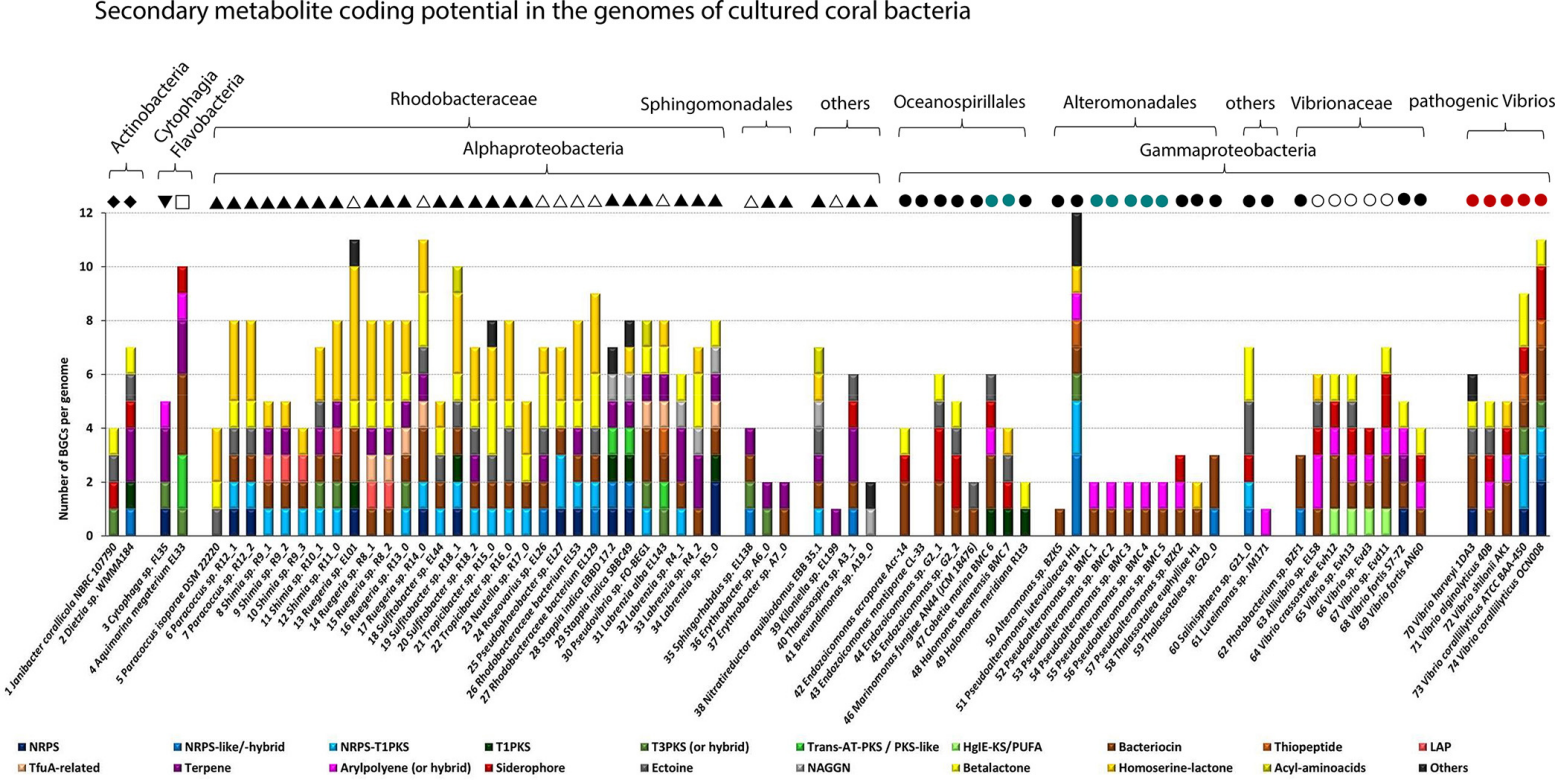
Distribution of biosynthetic gene clusters (BGCs) across 74 genomes of cultured coral bacteria. BGC counts per compound class were obtained using antiSMASH v.5.0 with default settings (and all extra features on). NAGGN, *N*-acetylglutaminylglutamine amide; LAP, linear azol(in)e-containing peptide; hglE-KS, heterocyst glycolipid synthase-like PKS; PUFA, polyunsaturated fatty acids; NRPS, nonribosomal peptide synthetase cluster; PKS, polyketide synthase cluster; TfuA-related, TfuA-related ribosomal peptides. The category “Others” comprises rare BGCs that had each less than three entries across the data set (among those were furan, ladderane-hybrid, phosphonate, polybrominated diphenyl ethers, lassopeptide, lanthipeptide, and butyrolactone BGCs). Symbol shapes above bars indicate the taxonomic class and the host origin of each genome (same as in Fig. 4).

We also assessed the secondary metabolite coding potential in the 74 genomes. AntiSMASH v.5.0 detected a total of 416 biosynthetic gene clusters (BGCs) across all genomes, whereby the number of BGCs varied substantially between strains, from no BGCs in Endozoicomonas montiporae CL-33 to 12 BGCs in Pseudoalteromonas luteoviolacea HI1 (Fig. 5). Bacteriocin clusters (*N *= 75), found in 81% of the strains, were the most frequently detected BGCs, followed by homoserine lactone (*N *= 62; in 43% of strains), nonribosomal peptide synthetase (NRPS; *N *= 59; in 51% of strains), beta-lactone (*N *= 46; in 53% of strains), terpene (*N *= 34; in 38% of strains), ectoine (*N *= 28; in 35% of strains), and siderophore (*N *= 25; in 28% of strains) clusters. The relatively large group of coral-associated *Rhodobacteraceae* genomes analyzed in this study presented a consistently rich BGC profile, characterized by the presence of bacteriocin, homoserine lactone, and NRPS-T1PKS clusters, while siderophore clusters were typically absent in this group. Siderophores were typically found in the *Vibrio* genomes of this study as well as in three of the four *Endozoicomonas* genomes. Characteristic for all *Pseudoalteromonas* genomes, including the BMC strains, was the presence of aryl polyene clusters, a compound class functionally related to antioxidative carotenoids (78). The absence of known BGC in the genome of E. montiporae CL-33 is an unusual outcome, as for example the closely related strains in the *Oceanospirillales* order usually display >4 BGCs (Fig. 5). The *E. montiporae* CL33 genome is complete (100% completeness, 0.9% contamination, 95.5% quality; 1 contig); hence, low assembly quality—which sometimes compromises the identification of large BGCs—does not explain the lack of BGCs in this genome.

## DISCUSSION

Here, we show that a taxonomically diverse array of bacteria can be isolated using a variety of medium and culture conditions. A total of 138 of these isolates (recruited from 52 studies) have been formally described, and at least 12 are putatively novel bacterial genera. It is promising that such extensive phylogenetic diversity can be captured from a limited number of culture media employed in the examined studies. Additional diversity is therefore likely to be captured through the implementation of alternative cultivation procedures that may improve our capacity to cultivate the “as-yet-uncultured” (28). Testimony to this is the observation that most of the strains assigned to the phylum *Firmicutes* in our meta-analysis were obtained almost exclusively from the various “custom media” utilized by different laboratories and blood agar alone, illustrating how diversification in cultivation design can widen the phylogenetic spectrum of the organisms isolated. In this regard, we anticipate that broader phylogenetic diversity will be gained within the culturable fraction if gradients in aerophilic conditions, temperature, and other physicochemical parameters are attempted along with innovative, less invasive techniques to extract microbial cells from the host matrix. The richness of bacterial phyla uncovered in this study corresponds to the phyla more often reported to dominate bacterial communities in corals by cultivation-independent studies (12), namely, *Proteobacteria*, *Bacteroidetes*, *Actinobacteria*, and *Firmicutes*, yet how diversity at lower taxonomic ranks within each phylum is captured remains to be determined. Another exciting challenge ahead is the unveiling of host-microbe and microbe-microbe molecular interdependence networks (e.g., cross-kingdom signaling and cross-feeding cascades) (79, 80). Such knowledge would likely enable laboratory captivation of so-far “unculturable” coral-specific or enriched lineages. Increasing the diversity of these coral-associated culturable bacteria will likely help in the identification of genomic features that could underpin the interaction with the host and its microbiome representing the foundation for experimental validation.

Although one of the initial aims of this study was to ascertain the percentage of culturable bacteria from a given coral species, it was deemed too speculative to report the findings due to variation in culture effort across the various studies. Indeed, this highlights the paucity of studies dedicated to determine exactly this, and there is a need for such mechanistic projects deploying multiple culture media and conditions to comprehensively sample bacterial associates from a single or a few host species. Collectively, studies aimed at capturing the culturable microbiome will extend our understanding of coral bacterial communities and their putative function in the coral holobiont. A catalog of cultures (as presented here and one which will hopefully be expanded) provides a means to increase our understanding of host-symbiote relationships. The ability to describe, understand, and culture specific symbionts from any given organism (like corals) also opens up the potential to utilize them as probiotics to restore degraded habitats (53, 61). For example, specific traits found in certain coral-associated bacteria, such as the presence of the genes *nifH* (nitrogenase), *nirK* (nitrite reductase), or *dmdA* (DMSP demethylase) involved in nitrogen and sulfur cycling, or those known to control pathogens, the enzymatic mitigation of reactive oxygen species (ROS) or other toxic compounds, may have roles in increasing coral health when the host is experiencing stress (53, 63, 81, 82). Identifying these traits via molecular analyses and laboratory tests using cultured bacteria with defined coral hosts will allow for the more rapid administration of native bacteria with the potential to help rehabilitate damaged corals. In addition, such a resource increases the possibility of identifying novel compounds of biotechnological interest (83). This seems particularly relevant in the case of coral-microbe symbioses, which are known to rank as one of the most prolific sources of bioactive molecules in the oceans (38).

A search in public databases (National Center for Biotechnology Information [NCBI]) found that, despite the 1,045 cultured coral-associated bacterial sequences with full-length 16S rRNA gene sequences, only 36 had genomes available as of February 2020. Clearly, a systematic effort to disclose the genomic features of coral-associated bacteria is needed in order to better understand the holobiont ecology and identify potentially beneficial microbes. As part of this study, we were able to add a further 38 to this tally (see Table S2 in the supplemental material). Even with this addition, the number of publicly available coral-associated bacterial genomes remains scant, and it is recognized that to more fully understand the roles of the cultivable fraction of coral bacteria, a thorough characterization of the species kept in culture, including genome sequencing, needs to be fostered alongside experimental biology and manipulative approaches. Moreover, a large collection of coral-associated genomes could also help to identify specific traits that are needed to thrive in the various niches within the hosts or point to those bacteria which offer a specific benefit to their host.

All of the available genomes were screened for an array of functions potentially important in establishing and maintaining interactions between bacterial symbionts and their marine invertebrate hosts. Overall, the *Endozoicomonas* and *Pseudoalteromonas* strains displayed high numbers of eukaryote-like protein-encoding genes important for host-symbiont recognition in well-studied systems such as marine sponges (65, 84, 85). The strain with the second highest number of eukaryote-like repeat protein-related entries (1,208 CDSs, after *Endozoicomonas* sp. G2_1 with 1,367 CDSs) was the octocoral associate *Aquimarina* sp. strain EL33 (class *Flavobacteria*). In the current culture collection, 15 additional *Aquimarina* isolates are reported, from the scleractinian corals Porites lutea, Pocillopora acuta, Stylophora pistillata, Acropora millepora, Acropora tenuis, and the octocoral E. labiata. Retrieving the genomes from these candidates will allow us to explore these emerging patterns in greater detail. For example, a recent comparative genomics survey of host-associated and free-living *Aquimarina* species revealed complex secondary metabolite biosynthesis and polycarbohydrate degradation capacities (86), but further investigation into their mechanisms of interactions with corals is warranted.

Only eight *Endozoicomonas* isolates (five of them type species) have so far been cultured from corals (according to our collated information). These are from the octocorals Eunicea fusca and *Plexaura* sp. and the scleractinian corals Montipora aequituberculata, Acropora cytherea, Acropora hemprichii, and *Acropora* sp. To date, only four of these (two from this study) have had their genomes sequenced (all from scleractinian corals) (18, 87). This is surprising given that numerous studies found that this genus is highly abundant in the healthy coral holobiont and seems to decrease in abundance upon deteriorating environmental conditions (e.g., reviewed in references 35, 88, and 89). Future cultivation efforts should therefore be directed toward the *Endozoicomonadaceae* family in order to increase the representation of their taxonomic and functional diversity in culture collections (29). In this regard, this study finds evidence that supplementing culture media with DMSP is an approach worth investing in future attempts to cultivate coral-associated *Endozoicomonas*, possible in combination with growth at lower temperatures (29). The metabolic data obtained from the comparative analysis of these four strains can be used, for example, to drive the selection of specific nutrients and conditions required to culture this particular genus of coral symbionts. Furthermore, there are 55 cultured *Pseudoalteromonas* strains in our collection which should also be explored regarding their symbiotic properties and their functional gene content (only 6 genomes currently available). Similar to *Endozoicomonas*, *Pseudoalteromonas* species are also frequent members of coral-associated microbiomes (35). A number of *Pseudoalteromonas* have been shown to display high antimicrobial activity, and many of these bacteria are isolated from coral mucus, lending support to the protective role the surface mucous layer has for the host and its importance in the coral holobiont’s defense—against bacterial coral pathogens in particular (90). Indeed, five of the six *Pseudoalteromonas* (where genomes are available) were shown to be effective BMCs when corals were challenged with the coral pathogen Vibrio coralliilyticus (53).

Having genomes available from the potential pathogens also allows for greater insight into coral biology, especially when interested in ascertaining pathogenicity-related traits (91, 92). For example, from the 11 *Vibrio* species for which genomic data were available, we were able to show functional separation (based on Pfam profiles) of known pathogenic and nonpathogenic strains. This was further accompanied by a significantly higher abundance of CDSs encoding for the type VI secretion system, important for virulence in the pathogenic strains (76). Prevalence of siderophore-encoding genes was also noted in the *Vibrionaceae* strains, suggesting that these bacteria likely gain competitive advantages through efficient and extensive iron acquisition, which is a trait often seen in opportunistic and pathogenic bacteria (93, 94). Hypothetically, the selection of beneficial microbes that are also good siderophore producers could add to the biological control of these pathogens. Indeed, two proposed BMC strains Cobetia marina BMC6 and Halomonas taenensis BMC7 harbor such siderophore clusters on their genomes and so did three of the four *Endozoicomonas* strains. However, the five *Pseudoalteromonas* BMC strains and the Endozoicomonas montiporae CL-33 had low numbers of BGCs, possibly indicating a reduced investment into secondary metabolism. Indeed, the low number of BGCs in these *Pseudoalteromonas* strains is in contrast to the established prevalence of biologically active compounds in many marine host-associated *Pseudoalteromonas* strains (95). In part, this may reflect a limitation of the software utilized to detect genes for all secondary metabolites, as genes for common metabolites (such as for the production of the antibiotic marinocin and those that produce tetrabromopyrrole coral larval settlement cues by *Pseudoalteromonas* [96, 97]) were not picked up. These bioinformatic limitations emphasize the importance of having bacterial cultures for the elucidation of the chemical ecology underpinning coral holobiont functioning.

Broader functional traits can also be ascertained from looking at the complete picture of isolates with annotated genomes. For example, 66% (49 out of 74) harbored the TauD gene, which is involved in taurine utilization (98). Two proposed BMCs, the Cobetia marina BMC7 and Halomonas taeanensis BMC7, revealed the highest copy number of TauD CDSs (seven and eight, respectively), while others range between one and five TauD copies. Taurine is an organo-sulfur compound widely present in animal tissues, and recent research has shown that obligate symbionts of sponges have enriched copies of taurine catabolism genes and taurine transporters in comparison with free-living bacteria (65, 72, 73). The widespread capability of the isolates studied here to potentially utilize host-derived taurine could guide the formulation of novel, taurine-containing cultivation media in the attempt to captivate coral symbionts, particularly from the important, yet underrepresented order *Oceanospirillales* (TauD was consistently present in all *Oceanospirillales* genomes [*N *= 8] analyzed here). The ubiquitous occurrence of bacteriocin clusters among the genomes is another example of broad-scale trends which we have identified in our genome meta-analysis. These may confer the specific culturable symbionts with particular competitive capacities toward closely related taxa in highly dense microbiomes (99, 100), as is commonly identified across corals and sponges. Moreover, the widespread presence of NRPS and beta-lactone clusters hints toward broad-spectrum antimicrobial and cytotoxic capabilities in multiple associates. It also corroborates the hypothesis that these marine metaorganisms are promising sources of novel bioactive compounds, representing targets for bioprospection (38). Many strains also possess homoserine lactone-encoding BGCs indicative of sophisticated, cell-density-dependent chemical communication mechanisms. Antioxidant activities are likely conferred by the presence of aryl polyene BGCs in the genomes (78, 101). These pigment type compounds, functionally related to carotenoids, characterized most of the proposed BMC strains. Furthermore, several coral-associated bacteria of different taxonomic origins are seemingly well equipped to handle osmotic stress as revealed by the occurrence of ectoine- and *N*-acetylglutaminylglutamine amide (NAGGN)-encoding genes. Therefore, there is a need to continue the effort in culturing coral-associated bacteria to explore new biosynthetic potentials, both for bioprospecting purposes and for better understanding the chemical ecology of the metaorganism.

Identifying likely candidates for symbiosis is one challenge, but once the candidates are confirmed and characterized, the need to understand how the animal host establishes symbiosis and retains the relationship will also be critical. However, this is a two-way street. Current research in sponges has revealed that bacteria expressing the ankyrin genes avoid phagocytosis by sponge amoebocytes, thus becoming residents of the sponge microbiome by evading the host's immune system (64, 70). Further, as ankyrin repeats are enriched in the microbial metagenomes of healthy corals (10, 11), it is expected that commensal coral-associated bacteria also use this aspect of ankyrin genes to establish symbiosis. The evolutionary forces shaping the symbiosis are even trickier here, as bacteriophages encode for ankyrin biosynthesis in their genomes and might transfer this information across different community members (70). As identified above with siderophore-encoding genes, similar patterns of symbiosis establishment and energy utilization may be adopted by both commensal and pathogenic bacteria.

To conclude, here we have highlighted that diverse coral-associated bacteria are already cultured, although these are often scattered across collections and rarely collated into one easily accessible location. Further, only a few of these have had their genomes sequenced. Despite the lack of genomes, we were able to identify a number of genetic features commonly encoded by these coral bacterial associates. These features include broad-spectrum antimicrobial, antioxidant, and cytotoxic compound production capabilities, high abundance of ankyrin repeat entries, tetratricopeptide, and WD40 repeats, and taurine degradation genes. That said, this can only be quantitatively assessed through comparison of metagenome profiles from corals versus other environments, such as sediments and seawater in a comprehensive fashion (several samples with replication, etc.). Such metagenome-based analyses should be complemented by (large-scale) marker gene surveys and/or visualization techniques to determine the nature and holobiont site of bacterial association, in particular since any metaorganism (configuration) is specific to a time and place and not static given the temporal (“fluidic”) nature of host-microbe interactions (102). Even though the statistical power, with only part of the representative genomes available from cultures (as in this study), is limited, we exemplify here the importance of the cultured bacterial fraction of corals in hypothesis testing and applied microbiology.

We end by highlighting the importance and need for a global initiative to create an online catalog of genomic and physiological features of cultured coral-associated bacteria. Combining the use of these genomic insights with innovative culturing techniques (37), aimed at improving the collection of coral-associated bacterial isolates, will see this field of coral biology move forward. Such an initiative should likely start with those microbes which have their complete genomes sequenced. This study pioneers the organization of such a global collection, as part of the efforts from the Beneficial Microbes for Marine Organisms network (BMMO), through a public invitation to researchers working in this field. As a result, we have here provided a list of cultured bacteria from corals that are currently available in public databases, plus isolates that were kept in collections from all the laboratories that responded to our invitation (Table S1 and available now, open access via http://isolates.reefgenomics.org). Now other researchers can access this virtual collection and/or contact specific laboratories for collaborations or solicitations of specific microbial strains.

## MATERIALS AND METHODS

### Literature search and data curation.

Google Scholar and the National Center for Biotechnology Information (NCBI) were searched for publicly available 16S rRNA gene sequences of cultured coral-associated bacteria (as of 2018). Search terms, including coral, bacteria, 16S, and culture, were utilized as well as combinations of these. The results were supplemented with data from culture collections from laboratories around the world through a public invitation to researchers working in this field. In total, we were able to obtain bacterial isolates originating from 84 coral species (representing tropical, temperate, and cold-water habitats) from all major oceans (Fig. 1; see also Table S1 in the supplemental material). Due to the number and varied nature of the different contributing sources of these isolates, parts of the associated metadata for certain cultures are missing or incomplete.

### Phylogenetic analysis and tree generation.

In total, we were able to collate 3,055 individual isolates which had (at least) part of the 16S rRNA gene sequenced (see Table S1 for details). We selected only high-quality sequences by removing those shorter than 500 bp, or longer than 1,600 bp and containing more than one ambiguity. Further, we utilized the mothur (v.1.42.0) commands screen.seqs and filter.seqs to remove poorly aligned sequences and positions without sequence information, respectively (103). This resulted in 1,045 isolates with near full-length 16S rRNA gene sequences, which were used in downstream phylogenetic analyses. To this end, sequences were aligned using the SILVA 138.1 database as a reference (104), and the clear-cut command was used within mothur to generate a phylogenetic tree using the relaxed neighbor-joining method (RNJ) (105, 106). To generate the distance matric, the default of percent identities (so-called p-distances) was retained.

A phylogenetic tree of coral species was also generated using the Taxonomy Common Tree tool of NCBI (107). Species names were added manually to create a tree file. Tree features were optimized using iTOL v4 (108).

### Taxonomic composition of bacterial isolates by medium.

Bacterial strains listed in Table S1 were sorted by isolation medium and subsequently grouped at phylum, order, and genus levels according to the current SILVA (138.1) taxonomy (104). Stacked column graphs, showing relative abundances of the cultivated taxa were created thereafter. At the genus level, all groups representing less than 1% of the total pool in each medium were included in a group labeled “others.”

### Genome analysis.

The integrated Microbial Genomes and Microbiomes database (IMG; https://img.jgi.doe.gov/) (109) from the Department of Energy’s Joint Genome Institute (DOE-JGI), and the assembly database from NCBI (https://www.ncbi.nlm.nih.gov/assembly) were searched for publicly available genomes from cultured coral bacteria in February 2020. Thirty-six bacterial genome assemblies (21 from scleractinian coral and 15 from octocoral associates) were downloaded from NCBI and included in this analysis. The annotation of genomic features such as genome size, GC content, and number of coding sequences (CDSs) was performed for all 74 genomes with the RAST server (110) (see Table S2). Protein families (Pfams) were predicted with the online server WebMGA (default settings) (111) using amino acid sequence files obtained from RAST. The resulting individual Pfam annotation files were then joined using a customized R script and the resulting count tables were Hellinger transformed for multivariate analyses (see Table S5). Dissimilarity between genomes based on the Pfam profiles were then calculated using the Bray-Curtis index. Ordination of the genomes based on their functional profiles was carried out using principal coordinate analysis (PCoA) and plotted in eigenvalue scale (i.e., scaling of each axis using the square root of the eigenvalue) with PAST software v3.25 (112). PERMANOVAs (permutational multivariate analyses of variance) were performed with 999 permutations to test for overall differences in functional profiles between bacterial genomes from different taxonomic classes. Five groups (classes) were used: *Alphaproteobacteria*, *Gammaproteobacteria*, *Actinobacteria*, *Cytophagia*, and *Flavobacteriia*. A separate PERMANOVA analysis of Bray-Curtis dissimilarities calculated for the 11 available *Vibrio* genomes was then performed in order to highlight differences between strains identified as potentially pathogenic (*N* = 5, group 1) and those apparently nonpathogenic (*N* = 6, group 2) (identification of pathogenicity from available literature—see references). Finally, AntiSMASH version 5.0 (113) was used with default parameters (and extra features “All on”) to identify biosynthetic gene clusters (BGCs) in all genomes.

### Data availability.

The newly described genomes associated with this project (38 in total; also see Table S2) can be found in the following BioProjects on NCBI: accession no. PRJNA698462, PRJNA638634, and PRJNA343499.

10.1128/mSystems.01249-20.1 FIG S1 Phylum (a), order (b), and genus (c) level profiles of coral-associated bacteria isolated from each coral species. Taxa (i.e., orders and genera) representing less than 1% of the total percentage of isolates were pulled together and classified as “Others.”FIG S1, JPG file, 0.6 MB Copyright © 2021 Sweet et al. 2021 Sweet et al. https://creativecommons.org/licenses/by/4.0/ This content is distributed under the terms of the Creative Commons Attribution 4.0 International license.

## Supplementary Material

Reviewer comments
