# Supplementary figures and images for: Insights into the Cultured Bacterial Fraction of Corals
Source: mSystems. 2021 Jun 22;6(3):e01249-20. doi: 10.1128/mSystems.01249-20 (PMC8269258; doi:10.1128/mSystems.01249-20)

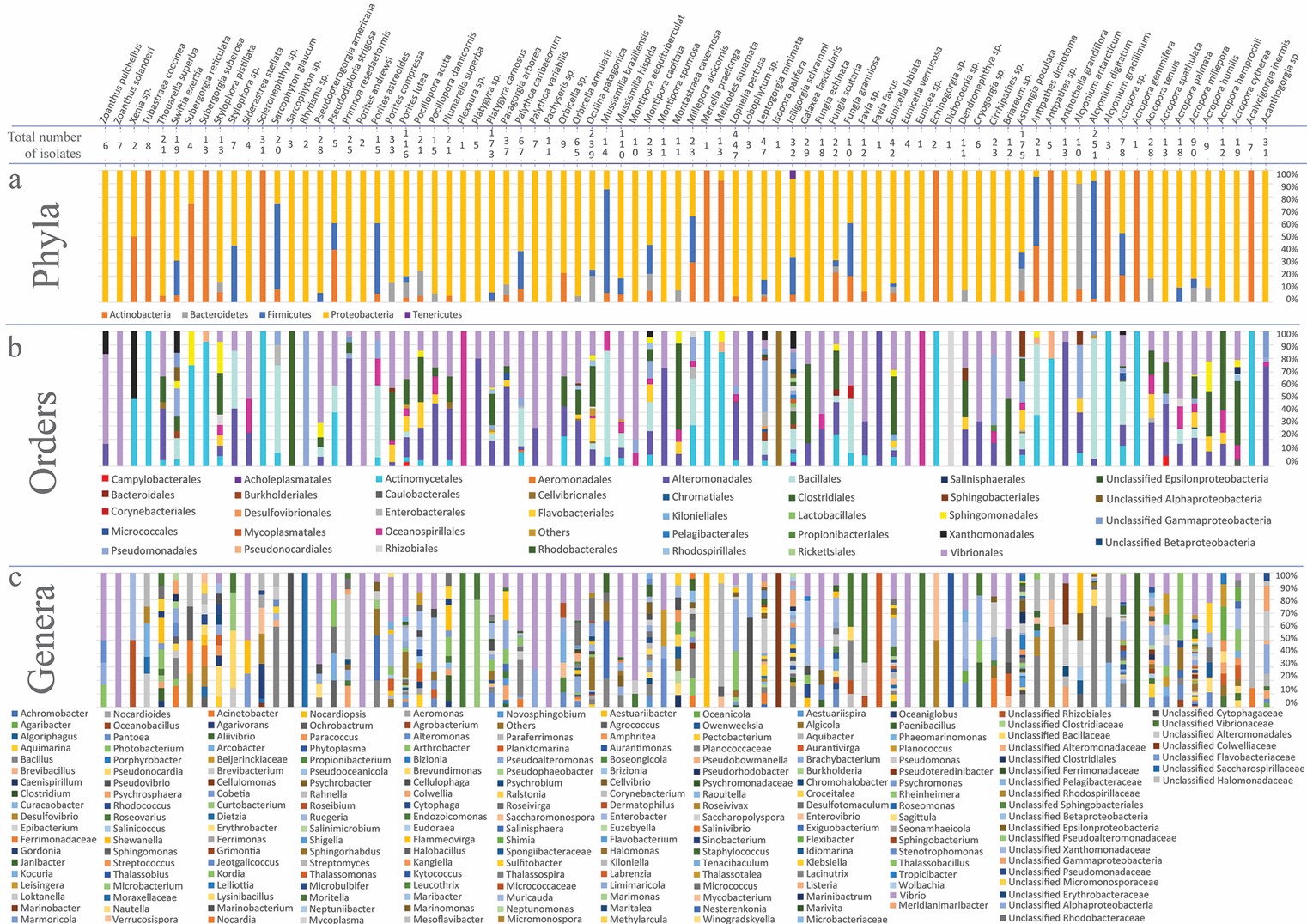

Supplement: FIG S1 [file msystems.01249-20-sf001.jpg]
